# Implicit and Explicit Contributions to Object Recognition: Evidence from Rapid Perceptual Learning

**DOI:** 10.1371/journal.pone.0047009

**Published:** 2012-10-08

**Authors:** Ulla Martens, Patricia Wahl, Uwe Hassler, Uwe Friese, Thomas Gruber

**Affiliations:** 1 University of Osnabrück, Institute of Experimental Psychology I, Osnabrück, Germany; 2 University Medical Center Hamburg-Eppendorf, Department of Neurophysiology and Pathophysiology, Hamburg, Germany; University of Montreal, Canada

## Abstract

The present study investigated implicit and explicit recognition processes of rapidly perceptually learned objects by means of steady-state visual evoked potentials (SSVEP). Participants were initially exposed to object pictures within an incidental learning task (living/non-living categorization). Subsequently, degraded versions of some of these learned pictures were presented together with degraded versions of unlearned pictures and participants had to judge, whether they recognized an object or not. During this test phase, stimuli were presented at 15 Hz eliciting an SSVEP at the same frequency. Source localizations of SSVEP effects revealed for implicit and explicit processes overlapping activations in orbito-frontal and temporal regions. Correlates of explicit object recognition were additionally found in the superior parietal lobe. These findings are discussed to reflect facilitation of object-specific processing areas within the temporal lobe by an orbito-frontal top-down signal as proposed by bi-directional accounts of object recognition.

## Introduction

Object recognition relies on the activity of cortically widespread networks which represent various stimulus features and which are distributed across different functional areas in the brain [1], [2]. These networks are also termed “cortical object representations” [3] and their activation is based on (A) implicit and (B) explicit mechanisms:

(A) Studies on ‘data-driven’ implicit influences on cortical object representations revealed that when an object is perceived repeatedly (and repetition is task-irrelevant), the object representation is tuned in a way that it becomes more effective in processing this stimulus [4], [5], [6], [7], [8], [9]. In particular, repetition-related effects were localized in occipital and temporal regions of the visual processing system, which supported the view of a bottom-up analysis within the ventral stream from simple feature detection to complex semantic and conceptual object representations [10].

(B) Explicit object recognition, i.e. the conscious perception of an object, was investigated by Bar and colleagues [11]. They demonstrated gradually increasing activity in the anterior temporal lobe with increasing confidence in an object's identity. In line with stimulus repetition studies, they concluded that a cortical hierarchy of object representations exists in the temporal lobe.

**Figure 1:**
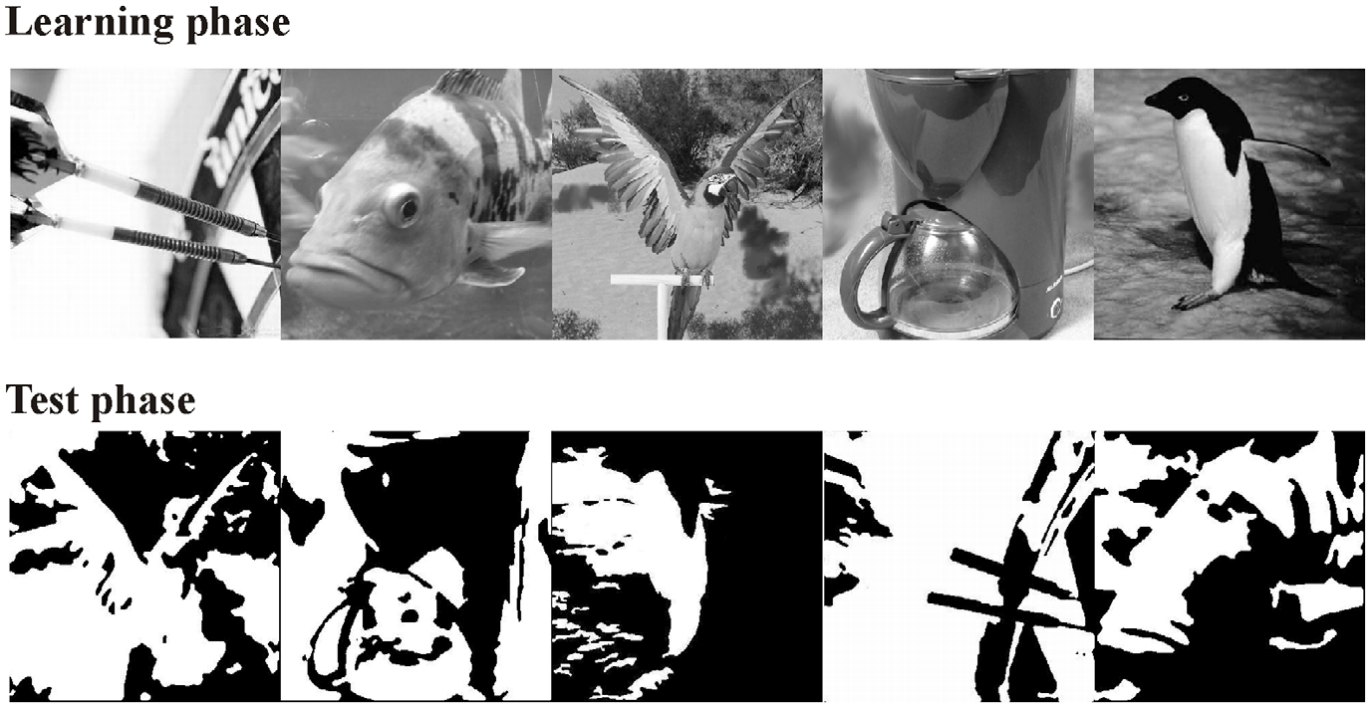
Experimental stimuli. Example stimuli of the original, gray-scale images used in the learning phase (living vs. non-living categorization) and of the corresponding degraded stimuli used in the test phase (recognized vs. not recognized)**.** Note that the order in the bottom row differs from the upper row to allow the reader to experience the initial meaninglessness of the degraded images.

Based on these results one can assume that cortical regions reflecting explicit and implicit object perception processes overlap to a certain degree. However, there is a lack of studies which replicated Bar et al.’s findings and which examine both processes within the same experimental design. To overcome this limitation we conducted an electroencephalogram (EEG) study and relied on a strategy from the field of memory research which was suggested by Rugg and his colleagues [12]. In an EEG study they confronted their participants with a series of words which were either presented for the first time (new words) or with words which were previously presented during an incidental learning phase (old words). Rugg et al. demonstrated that old words, which were correctly classified as being old, triggered explicit processes. Old words erroneously classified as being new, elicited implicit processing. To adopt this design for our purposes we proceeded as follows: During an incidental learning phase participants were confronted with gray-scale images of everyday objects. Subsequently, degraded versions of some of these images were presented intermixed with degraded versions of new images (see Figure 1 for examples). Hereby, some of the degraded pictures can be explicitly recognized based on the previous exposure to the corresponding original images (a phenomenon also known as ‘rapid perceptual learning’; see e.g. [13], [14], [15]). Subsequently, specific electrophysiological markers (see below) were contrasted: the contrast between successfully recognized *versus* new objects, which should reveal the object recognition network to which both implicit and explicit processes contribute. Implicit perceptual processes to incidentally learned stimuli that are not consciously recognized will be uncovered by the contrast between unrecognized *versus* new objects [12]. The contrast between recognized *versus* unrecognized objects will reflect the activation of explicit processes that allow for conscious perception. Although calculating the identical contrast as Rugg and colleagues [12], we assume - but do not know - that implicit and explicit processes contributing to object recognition are similar but not identical to implicit and explicit memory processes. Our concept of “implicit” perceptual processes refers to pure data-driven, automatic perceptual analysis of the sensory input, which is independent of successful recognition. Thus, we suppose that prior exposure to the original image (a) triggers early, low-level perceptual processing of the fragmented version (i.e. automatic, implicit, without conscious recognition), and (b) increases the probability of consciously recognizing the fragmented object (i.e. explicit retrieval of the object representation).Experiments relying on the rapid perceptual learning of degraded pictures have the advantage that the physical stimulus parameters of recognized and unrecognized objects are highly comparable (i.e. the only difference is recognition performance). However, the stimulus material is difficult to construct and therefore limited. Thus, one has to deal with relatively low trial numbers, a problem for reliable EEG analysis and in particular for robust source reconstructions. To overcome this problem we applied the so-called steady state visual evoked potential (SSVEP) technique. The SSVEP is the oscillatory response of the brain to a flickering stimulus in the same frequency as the initiating stimulus [16]. The ongoing (i.e. steady) oscillatory response is characterized by a good signal-to-noise ratio which reveals reliable results even with a limited amount of trials [17]. In a series of previous studies it was demonstrated that SSVEP modulations are sensitive to successful object recognition [18], implicit mechanisms underlying object recognition [9] and mnemonic functioning [17], [19].

To summarize, the present study intends to investigate implicit and explicit perceptual processes of rapidly learned objects by means of SSVEPs (in combination with EEG source reconstructions). We hypothesize that explicit object recognition will modulate the neuronal activity within a cortically widespread object representation. Furthermore, we expect implicit processes to occur within this network, even in the absence of successful object recognition.

## Methods

### Ethics Statement

The study conformed with the Declaration of Helsinki and was approved by the ethics committee of the University of Osnabrück. Participants took part after giving written consent.

### Participants

Twenty healthy participants took part in the experiment. The data of two participants had to be excluded from the analysis due to technical problems during the recording. The remaining eighteen participants had an average age of 28.6 years (15 female, 2 left-handed by self report) with normal or corrected-to-normal vision.

### Stimuli and Procedure

The stimulus-set consisted of 250 gray-scale photographs of living (e.g. dog, tulip) and non-living (e.g. house, cup) objects. Degraded (i.e. binarized) versions of 200 of these gray-scale stimuli were created by designating gray values below a certain threshold to black and values above this threshold to white. The aim was to create stimuli that would not be recognized when seen for the first time but after the original image had been revealed. Figure 1 shows example stimuli for the original and the degraded images. The degraded stimuli were tested in two behavioral studies (16 participants in each study) with regard to recognizability. Average performance to the final stimulus set was 13% (SD = 7%) recognition (i.e. they could explicitly state the name of the displayed object) of degraded images during their first presentation and 53% (SD = 13%) were recognized after the original image had been revealed.

Each block of the present study consisted of two phases: an incidental learning phase and a test phase. During the learning phase 15 original images were presented of which five were distractor images (i.e. they were not presented as degraded version later). The displayed objects had to be classified as living or non-living by key press. Subsequently in the test phase, degraded versions of ten of these learned original images were presented together with degraded pictures of ten unlearned (i.e. new) images. Participants had to state by key press whether they recognized the degraded object or not. ‘Recognized’ was defined as being able to name the object. Stimulus-response assignments during learning and test phase were counterbalanced across participants. In total ten blocks were presented, resulting in the classification of 100 learned fragments and 100 unlearned fragments. After each block a break was provided.

Each trial in the learning phase consisted of the presentation of a fixation cross for 500 ms and the subsequent display of the object stimulus (7×7° visual angle) at the center on a black background. The stimulus remained on the screen until the participant indicated whether the object was living or not. After one second the next learning trial started.

During the test phase, each trial started with the presentation of a fixation cross for 500 to 900 ms. Subsequently, a degraded stimulus (7×7° visual angle) was presented for 3000 ms at 15 Hz synchronous to the screen refresh rate of the 60 Hz monitor. After the stimulus disappeared, participants were prompted to indicate whether they did or did not recognize an object. Five hundred milliseconds after giving the response with the middle or index finger of the right hand, the next test trial started. In 20% of the test trials a magenta-colored dot was superimposed on the degraded stimulus for 67 ms at a random position around the centre of the picture. These trials were introduced to uphold attention to the stimuli during the presentation period. Participants’ task was to press immediately the space bar when they detected the dot that could appear between 100 and 2700 ms after stimulus onset.

After this experiment participants performed an unannounced naming task. Specifically, all degraded images that were classified as “recognized”, were presented again, each for 1300 ms and participants had to type for every image the name of the object they recognized.

### Electrophysiological Recordings

During the test phase, participants’ electroencephalogram (EEG) was recorded by a 128 electrode set up and the BioSemi Active-Two amplification system (sampling rate 512 Hz). The recording took place in an electrical shielded room. Eye movements and blinks were controlled by vertical and horizontal electrooculogram (EOG). Two additional electrodes served as reference and ground (*CMS: Common Mode Sense* and *DRL: Driven Right Leg*).

Artifact correction was performed offline with *Statistical Correction of artifacts in dense array studies* [20]. Single epochs with excessive eye movements and blinks or more than 20 channels containing artifacts were discarded from further analyses. The EEG was segmented into epochs from −500 to 3000 ms relative to the onset of the degraded stimulus (baseline −200 to 0 ms) and the data were re-referenced to the average of all electrodes. Target trials (i.e. trials containing a magenta dot) were excluded from analyses.

### Data Analysis

#### Behavioral data

Response accuracy was measured in the learning phase (living vs. non-living categorization), in the test phase (recognized vs. not recognized), for the target detection in the test phase as well as for the answers provided in the naming task.

#### SSVEPs in electrode space

**Figure 2:**
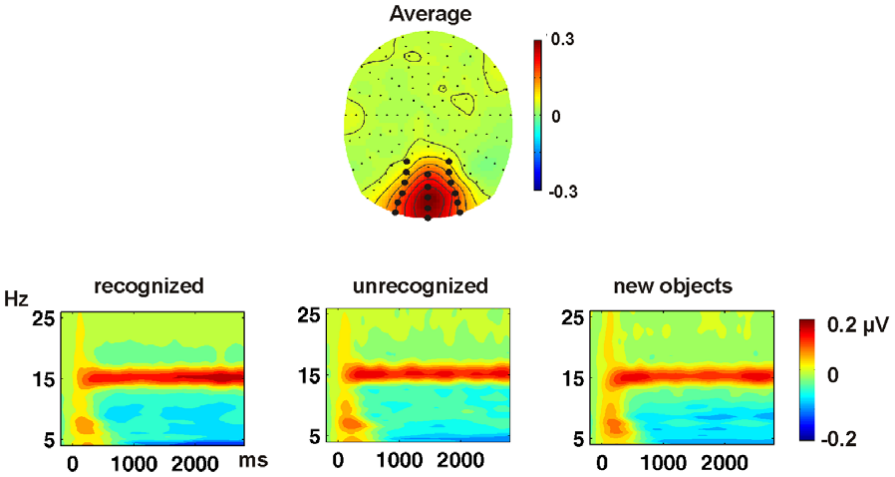
The 15 Hz SSVEP. Topography of the SSVEP averaged across all conditions in the time-window 750 to 2500 ms. Time by frequency plot across occipital electrodes (marked in the topography plot) separately for the three analyzed conditions. A clear SSVEP at 15 Hz can be appreciated in all conditions.

To determine the temporally changing magnitude of the SSVEP at 15 Hz, the signal was spectrally decomposed by means of Morlet wavelet analysis as described in previous studies [18]. To validate that a 15 Hz SSVEP was elicited, we plotted a time by frequency representation from 1 to 30 Hz separately for all conditions (see Figure 2). For statistical analyses the spectral decomposition related to the 15 Hz wavelet was used. The final data set consisted of SSVEP amplitude values elicited by fragmented stimuli that were presented as original during the learning phase and recognized (in the following referred to as *recognized*), stimuli that were previously presented but not recognized (referred to as *unrecognized*) and stimuli that were not previously presented (i.e. not learned) and not recognized (referred to as *new* objects).

Trials with pictures that were not learned but recognized were not analyzed, because these were rare events (see Table 1). Trial numbers in the other three conditions were equalized by omitting the latest events of conditions with a larger number of trials. To determine the topographical activation differences between conditions, statistical comparisons were carried out by means of paired t-tests of the averaged amplitude values in the time-window from 750 to 2500 ms across 17 posterior electrodes at which the SSVEP was largest (see Figure 2). This time-window was chosen according to previous studies and to eliminate an overlap of SSVEPs and conventional ERPs [21], [22], [23].

**Table 1:** Responses were categorized according to whether a stimulus was learned in the living/non-living task and recognized in the test phase.

Test phase	Learning Phase		
		incidentally learned	new
	recognized	**(A) successful recognition** (implicit andexplicit processes)	**(B)** -not analyzed-
	not recognized	**(C)no recognition** (implicit perceptualprocesses)	**(D) no recognition**

Cell A refers to successful recognition, which is served by implicit and explicit processes. Cell C lacks the explicit recognition process; however implicit perceptual processes should occur due to the repeated exposure to the learned stimulus. Cell D refers to new stimuli that had not been seen before and were not recognized in its degraded form.

Conducted contrasts were A vs. D, reflecting successful object recognition; A vs. C reflecting explicit recognition aspects, and C vs. D reflecting implicit perceptual processes.

#### SSVEPs in source space

The cortical sources of SSVEP effects were localized with VARETA (Variable Resolution Electromagnetic Tomography; Bosch-Bayard et al., 2001). This procedure provides the spatially smoothest intracranial distribution of current densities in source space which is most compatible with the amplitude distribution in electrode space [24]. The SSVEP was transformed into the frequency domain as described above (wavelet analysis) and VARETA was applied to the complex wavelet coefficients. The inverse solution consisted of 3244 grid points (‘voxels’) of a 3D-grid (7 mm grid spacing). This grid and the arrangement of 128 electrodes were placed in registration with the average probabilistic MRI brain atlas (‘average brain’) produced by the Montreal Neurological Institute (MNI; [25]). To localize the activation difference between the conditions in the time-window of 750 to 2500 ms, statistical comparisons were carried out by means of paired t-tests. All statistical parametric maps were thresholded at p<0.01. The outcomes were depicted as slices in the transversal plane constructed on the basis of the MNI average brain.

## Results

### Behavioral data

In the learning phase, participants categorized living and non-living objects correctly in 98% of the trials. In the test phase, participants recognized 44% (SD = 17%) of the previously learned objects and 86% (SD = 12%) of new objects were not recognized. Target detection was on average successful in 98.8% of the test trials.

The correctness of the answers in the subsequent naming task was 70%. We are convinced that this percentage provides a conservative measure of participants’ actual recognition performance during the test phase. The time delay between learning and test was much shorter (2 min) than the delay between test phase and naming task (approximately 30 to 45 min). Thus, it is likely that in the test phase some stimuli were recognized that were not recognized in the naming task. This assumption was confirmed by the participants’ subjective reports.

### SSVEPs in Electrode Space

**Figure 3:**
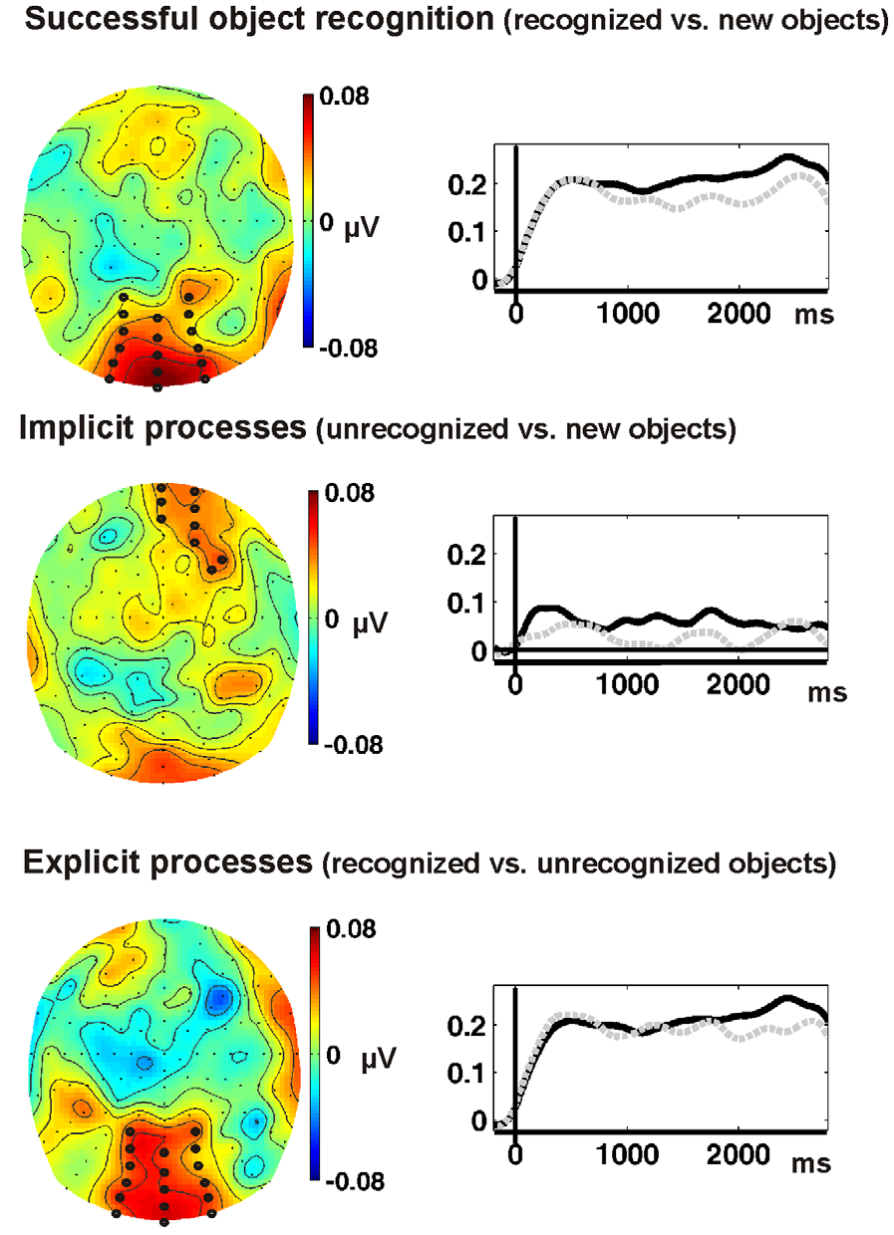
SSVEP effects in electrode space. Top row: SSVEP difference topography (A minus D, *cf.* Table 1) averaged in the time-window of 750 to 2500 ms and time-course of the SSVEP amplitude at significant occipital electrodes (marked in the topography) to recognized (solid line) and new objects (dashed line). Middle row: SSVEP difference topography (C minus D) averaged in the time-window of 750 to 2500 ms and time-course of the SSVEP amplitude at significant frontal electrodes (marked in the topography) to unrecognized (solid line) and new objects (dashed line). Bottom row: SSVEP difference topography (A minus C) averaged in the time-window of 1800 to 2500 ms and time-course of the SSVEP amplitude at significant occipital electrodes (marked in the topography) to recognized (solid line) and unrecognized objects (dashed line).

Figure 2 shows that in every condition of the test phase SSVEPs were elicited at 15 Hz with the typical maximum at occipital electrodes. Successful object recognition was reflected by significantly higher SSVEP amplitudes at these posterior electrodes for recognized objects as opposed to new objects in the time window from 750 to 2500 ms, t(17) = 2.67, p<0.016. Comparing the SSVEPs to unrecognized objects with new objects (i.e. reflecting implicit perceptual processes), revealed no significant occipital (t<1) but frontal differences, t(17) = 3.2, p<0.005. The comparison of SSVEPs to recognized objects with SSVEPs to unrecognized objects showed that explicit recognition processes affected the SSVEP first in the time window from 1800 to 2500 ms, t(17) = 2.17, p<0.044. The topography and time course of these effects are displayed in Figure 3.

### SSVEPs in Source Space

**Figure 4:**
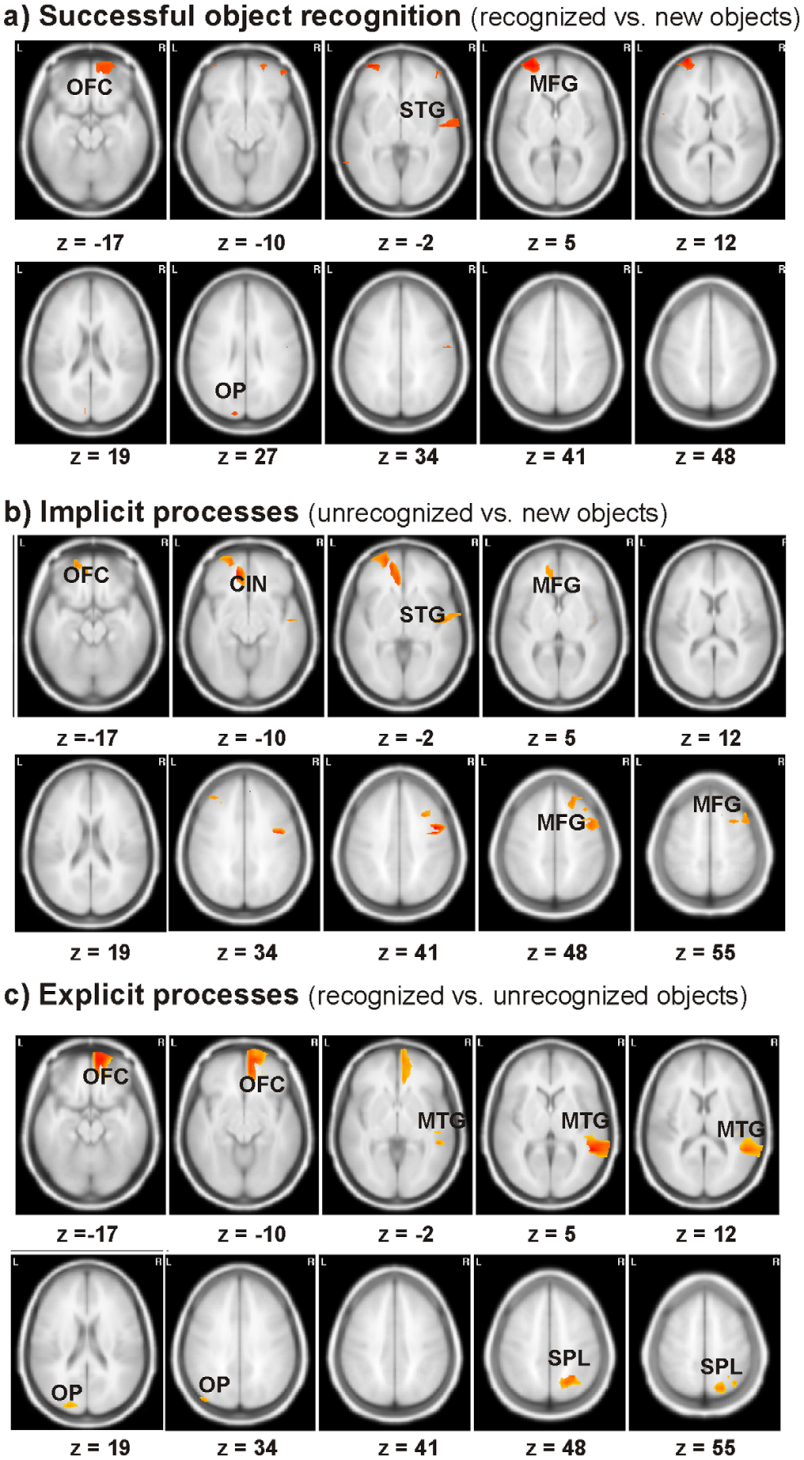
Source localizations of the SSVEP effects. a) A minus D, b) C minus D and c) A minus C with red indicating activations (p<0.01), MNI plane coordinate, left is left. OFC = orbito-frontal gyrus, CIN = cingulate region, MFG = middle frontal gyrus, STG = superior temporal gyrus, MTG = middle temporal gyrus, SPL = superior parietal lobe, OP = occipital pole.

The contrast in the time window of 750 to 2500 ms between recognized *versus* new objects revealed significantly stronger activations to recognized objects in left middle frontal, left middle temporal and superior occipital regions as well as in right orbito- and inferior frontal, right superior temporal and parietal areas. The activated sources are specified in Table 2 and visualized in Figure 4. Defining the cortical areas of implicit perceptual processes by calculating the contrast between unrecognized *versus* new objects revealed stronger activations bilaterally in the frontal cortex, in the left orbito-frontal cortex and in the right superior temporal gyrus. Comparing activations to recognized objects with unrecognized objects in the time-window of 1800 to 2500 ms revealed that these explicit recognition aspects were generated in the right orbito-frontal gyrus, the right middle temporal gyrus, the right superior parietal lobe and the left middle occipital gyrus. Deactivations did not reach statistical significance in any contrast.

**Table 2:** Brain areas identified by the three conducted contrasts. MNI coordinates of the activation peaks are provided.

Effect		Location	x	y	z
**Object recognition**	recognized >new objects	left middle frontal gyrus**	−31	60	5
		left middle temporal gyrus**	−60	−55	−2
		left superior occipital gyrus**	−13	−90	27
		right orbito-frontal gyrus**	18	56	−17
		right inferior frontal gyrus**	43	53	−10
		right superior temporal gyrus**	58	9	−2
		right postcentral gyrus**	57	−10	34
		right superior parietal lobe*	23	−63	48
**Implicit processes**	unrecognized >new objects	right middle frontal gyrus**	28	9	55
		right superior frontal gyrus**	29	17	63
		right superior temporal gyrus**	45	−11	−2
		left orbito-frontal gyrus*	−21	55	−17
		left middle frontal gyrus**	−22	61	−2
		left cingulate**	−13	40	−10
**Explicit processes**	recognized >unrecognized	right orbito-frontal gyrus**	10	60	−17
		right middle temporal gyrus**	53	−45	5
		right superior parietal lobe*	26	−58	48
		left middle occipital gyrus*	−45	−83	27

* p<0.05, ** p<0.01.

## Discussion

This study investigated implicit and explicit processes subserving object recognition by measuring SSVEPs in a rapid perceptual learning design. During an incidental learning phase participants categorized object images into living/non-living. In the subsequent test phase degraded images of incidentally learned and new objects were presented. Participants indicated whether they recognized an object or not. Behavioral results revealed that approximately half of the incidentally learned stimuli were recognized, which enabled analyses across equal trial numbers and thereby the localization of SSVEP effects reflecting successful object recognition, implicit perceptual processes, and explicit recognition processes.

The results of our study indicate that implicit and explicit processes contributing to object recognition overlap to a large degree and modulate activations within the widespread object recognition network that comprised frontal, temporal, parietal and occipital regions (contrast A vs. D) similar to previous studies [2], [9]. In contrast, Rugg and colleagues demonstrated that implicit and explicit memory processes seem to be distinct to a large degree [12]. Investigating the differences between memory and perception related implicit and explicit processes will be the aim of a further study.

While modulations by implicit perceptual processes (contrast C vs. D) were limited to frontal and temporal regions, the isolated explicit recognition process (A vs. C) additionally activated superior parietal regions. The parietal lobe is considered to play a substantial role in spatial attention to the constituting features of an object and in successful feature binding [26], [27]. Considering the specificity of the used stimuli as well as the task requirements, object recognition should have been highly dependent on successful feature binding. Alternatively, memory research discusses the posterior part of the parietal cortex to play a role in episodic memory retrieval [28], [29]. Thus, recollecting the event from the learning phase could have likewise yielded successful object recognition. Schott and colleagues, who compared explicit and implicit memory processes in a stem completion task, also reported activation of the superior parietal lobe when subtracting priming related activity from recognition memory [30]. They discussed this finding as evidence for a possible co-occurrence of implicit and explicit memory processes (see also [12]). Our results extend these findings to object recognition.

In contrast to superior parietal regions, the temporal lobe seems to play an important role in perceptual processes even without explicit recognition. Such gradual activation would be in line with the results of Bar and colleagues, who demonstrated that with increasing awareness of an object’s identity cortical activations within the temporal lobe intensify and shift anteriorly [11]. Comparing implicit perceptual processes with explicit recognition processes in our study revealed a shift from superior temporal gyrus to the middle temporal gyrus with conscious object recognition. Whereas the superior temporal gyrus more likely contains object-specific representations [21], the middle temporal gyrus has been discussed to reflect the activation of conceptual object representations [8], [21], [31]. Thus, it seems that the difference between successfully recognized and unrecognized degraded objects lies either in successful feature binding or in the retrieval of the learning event (i.e. superior parietal lobe activation) that allows activating higher-order object representations. The strong frontal locus of activations in the unrecognized trials could reflect coping mechanisms that try to solve the conflict between activated object representations in the temporal lobe and the lack of binding or retrieval cues [11]. Such an interpretation would also explain the lack of such frontal activations in repetition priming studies using clearly defined recognizable and unrecognizable objects [9] and the stronger frontal activations observed in the SSVEP topography for implicit perceptual processes as opposed to the topography reflecting successful object recognition.

The modulations of the orbito-frontal gyrus (OFC) observed in all three contrasts have been previously discussed to play a fundamental role within the object recognition network [32], however they have not been reported in repetition priming studies before [6], [9], [33]. The OFC is discussed to generate expectations and to predict the content of the visual input and thereby limit the amount of potentially relevant object representations [32]. Specifically, Bar and colleagues propose that coarsely analyzed visual input is directly projected from visual cortex to the OFC. Here, a cortical signal is initiated that propagates top-down to temporal cortices, in which the predictions are then integrated with the results of the more detailed but slower bottom-up process within the ventral stream. The stimuli in our study provided ideal input for the OFC due to their large amount of low-spatial frequencies. The visual cortex projects via the dorsal stream low-spatial frequencies to the prefrontal cortex [34], and the OFC seems to respond more selectively to low rather than high spatial frequencies [32]. We assume that in our study the OFC provided a best guess for figure-ground segmentation [35] as it was stronger active to incidentally learned as opposed to new objects [32], [36] and additionally stronger activated in response to recognized as opposed to unrecognized objects. This OFC signal subsequently limited the number of activated object representations in the temporal lobe and thereby increased the probability of successful object recognition. Support for this top-down information flow comes from a study by Bar and colleagues, who reported a 50 ms advantage for occipital-OFC phase-locking over OFC-fusiform phase-locking [32]. Importantly, we do not suggest that object recognition is only achieved by top-down processes. However, we assume that degraded stimuli are more likely processed via a top-down than a bottom-up route, which explains the strong frontal activations in this study. Thus, applying such stimuli opens further possibilities to investigate top-down accounts of object recognition in more detail.

Taken together the present study found indications that implicit and explicit processes contribute to object recognition within largely overlapping brain regions, with explicit processes additionally modulating the superior parietal lobe. Additionally, we assume that object recognition of degraded, low-spatial frequency images, as used in this study, is partly achieved by top-down facilitation of object-specific processes within the temporal lobe. On the basis of studies analyzing neuronal coupling [e.g. 32] it can be speculated that this top-down signal might originate from orbito-frontal cortex thereby supporting the assumptions of bi-directional object recognition accounts.
